# Parental Marital Quality and School Bullying Victimization: A Moderated Mediation Model of Parent–Child Attachment and Child Gender

**DOI:** 10.3390/children12070825

**Published:** 2025-06-23

**Authors:** Guojie Peng, Qiwen Liang, Siying Li, Xin Li, Weiqi Mu, Mingjie Zhou

**Affiliations:** 1 Institute of Psychology, Chinese Academy of Sciences, Beijing 100101, Chinazhoumj@psych.ac.cn (M.Z.); 2 Department of Psychology, University of Chinese Academy of Sciences, Beijing 100049, China; 3 Department of Student Affairs, Shanghai Communications Polytechnic, Shanghai 200030, China

**Keywords:** parent–child attachment, school bullying victimization, parental marital quality, gender, moderated mediation, children

## Abstract

**Background/Objectives**: School bullying is a significant issue that negatively impacts children’s well-being, emphasizing the need to identify family-related factors contributing to bullying victimization. This study explored the potential link between parental marital quality and school bullying victimization by employing a moderated mediation model. **Methods**: Parent–child attachment, measured separately as father–child and mother–child attachment, was tested as a mediator, with child gender included as a moderator. Data were collected from both children and their mothers, comprising 358 mother–child pairs recruited from three primary schools in suburban Beijing, China. **Results**: Results revealed that greater parental marital quality was associated with a lower risk of bullying victimization, with father–child attachment mediating this relationship. Furthermore, child gender moderated the mediating effect of father–child attachment, such that the indirect pathway from parental marital quality to bullying victimization through father–child attachment was statistically significant for girls but not for boys. **Conclusions**: These findings highlight the importance of father–child attachment in preventing bullying victimization and suggest that gender-sensitive implications may be necessary.

## 1. Introduction

Early socialization of children depends much on their family environment, which greatly shapes their psychological and behavioral growth [1]. Parental marital quality, the main element of the family surroundings, has become clear as a key predictor of children’s mental health and behavior [2]. Recent studies have demonstrated a growing concern about bullying victimization in schools, which has substantial and long-term consequences for victims’ psychological well-being [3,4,5]. The importance of family dynamics in shaping a child’s vulnerability to bullying and other negative social experiences cannot be ignored. That means it is critical to investigate the correlation between parental marital quality and bullying victimization. This understanding is beneficial in both its theoretical and practical applications.

### 1.1. Parental Marital Quality and School Bullying Victimization

Marital quality refers to both interpersonal aspects, like intimacy, consensus, agreement, sexuality, harmony, conflict, and disagreements, as well as intrapersonal aspects that include pleasure and happiness [6]. These features of parental marital quality significantly shape children’s psychological development [2]. Frequent conflict and a lack of support define low marital quality, which causes parents to get frustrated and then irritable, impatient, and emotionally exhausted [7,8]. This condition threatens children’s emotional security, hence raising their risk of anxiety, depression, and behavior problems [1,9]. Children from families like these sometimes struggle with social contacts and emotional regulation [10]. These results emphasize the significance of parental marital quality as a main determinant of mental health. Therefore, parental marital quality can be viewed as a protective factor for children.

Bullying is a hostile and proactive form of aggression that includes both direct and indirect behaviors, repeatedly targeting individuals or groups perceived as weaker [11]. Negative peer relations, including school bullying, have a profound impact on children’s mental health [12]. Bullying can cause lasting impacts on victims [4], frequently resulting in significant psychological distress, including anxiety, depression, loneliness, and low self-esteem [13]. Victims of bullying are more likely to experience internalizing problems, struggle academically, and engage in self-harm [4]. Moreover, some studies have shown that suicidal thoughts and attempts are significantly predicted by bullying victimization [14,15,16].

Numerous studies have investigated the correlation between parental marital quality and children’s psychological well-being; however, fewer have focused on victims of bullying. According to family systems theory, the family works as an integrated system, with the parents’ marriage influencing many elements of family dynamics [17]. High marital quality fosters increased family intimacy and adaptability, which in turn enhances the emotional security of children and mitigates the emergence of internalizing issues. For example, Sevda and Sevim (2012) discovered that students from healthy family environments were less likely to be involved in bullying, including as victims, than those from dysfunctional families [18].

In addition, social learning theory posits that parents, who serve as the primary role models for their children, exert an influence on their behaviors through their actions and interactions [19]. The behaviors parents display serve as models for children, who are likely to adopt similar conflict resolution strategies when facing peer conflicts. This modeling of maladaptive conflict resolution strategies influences children’s vulnerability to bullying victimization, as they may replicate these behaviors in their interactions with peers. Accordingly, we hypothesize that.

**H1:**  *Parental marital quality is negatively correlated with bullying victimization.*

### 1.2. The Role of Parent–Child Attachment in the Link Between Parental Marital Quality and Bullying Victimization

Attachment theory underscores the significance of early parent–child relationships in the formation of individual psychological development. The emotional bond that develops between children and their parents, known as parent–child attachment, is characterized by intimacy and enduring support. This bond provides children with a sense of emotional stability and security [20]. This attachment relationship lays the foundation for an individual’s internal working model, influencing self-worth, capabilities, and patterns of interpersonal interactions [21]. Consequently, the secure parent–child attachment has profound effects on individual development, influencing not only emotional regulation but also social skills [22].

School bullying, as a typical negative peer relation [12], can be influenced by parent–child attachment. Secure parent–child attachment is a strong predictor of positive peer relationships, which are crucial for school adjustment [23]. Children with secure attachments are stronger in the areas of fairness, forgiveness, humor, and kindness, all of which are interpersonal strengths and make it easier for them to create friendships of a higher quality [24]. Peer support, which is linked to friendship, is crucial in physical education to prevent abuse [25]. Furthermore, secure attachment improves children’s emotional stability and coping skills, both of which are critical for successfully handling peer conflicts [22]. Militsa et al. (2013) discovered that children’s engagement in bullying, either as victims or perpetrators, is significantly predicted by their parents’ low-quality bonds [26]. The findings demonstrated how important parent–child attachment is in determining how children interact with others and how susceptible they are to bullies. Furthermore, a long-term study showed that positive parent–child relationships contribute to protecting children from ongoing bullying victimization and greatly decrease the likelihood of psychological distress [27]. The study revealed that parental support plays a direct compensatory role by buffering the negative effects of bullying.

Recognizing the distinct roles that both mothers and fathers play in children’s development is essential. Different cultures allocate childcare responsibilities differently, but most societies agree that mothers normally perform the key role in childrearing [28,29]. In contemporary parenting science, the idea of father “involvement” has received emphasis, emphasizing fathers’ involvement, accessibility, and responsibility in their children’s development [30]. While mothers are frequently the primary caretakers and socializers for their children, helping them develop emotionally and socially [31], fathers play a special role in their children’s autonomy development. Fathers, as opposed to mothers, are more likely to encourage competitiveness, independence, and risk-taking [32]. Importantly, fathers’ contributions to child development are distinct from those of mothers. Research has shown that both fathers’ and mothers’ supportive parenting separately predict children’s language and cognitive development [33]. Given the different influences of mothers and fathers on child development, we chose to separate father–child and mother–child attachment in our hypotheses to investigate potential differences. Consequently, we propose that.

**H2a:**  *Mother–child attachment is negatively related to bullying victimization.*

**H2b:**  *Father–child attachment is negatively related to bullying victimization.*

In addition to the direct impact of parent–child attachment, recent research has improved our comprehension of the mechanisms through which parental marital quality influences this attachment, thereby disclosing the extent to which family relationship quality influences the developmental outcomes of children. Beginning with the establishment of the marital relationship, the marital subsystem serves as the foundation for the overall dynamics of a family system. In turn, the parent–child subsystem is influenced by this marital subsystem through mechanisms such as the compensation or spillover hypotheses [34].

According to the spillover theory, emotional and behavioral variations within the marital subsystem might directly or indirectly affect children’s developmental paths by flowing into the parent–child subsystems [29]. Various studies have confirmed this [35,36,37]. For instance, Kuo (2022) showed by a spillover effect that parent–child emotional climates are much impacted by marital quality [35]. In particular, variations in marital emotional states significantly impact the emotional dynamics between parents and children, therefore influencing children’s social and emotional growth. The research underscored the critical role of marital stability in the development of children’s emotional well-being and behavior, emphasizing that positive marital qualities contribute to more effective emotional regulation within the parent–child relationship. Conversely, in marriages that are characterized by frequent conflict and emotional distress, the negative emotions and tensions between parents commonly spill over into the parent–child relationship. Such situations may exacerbate conflict between parents and children, promoting cognitive appraisal and emotional insecurity, which subsequently predicted increased depression and social anxiety in children [38]. Additionally, conflicts are associated with children’s internalizing and externalizing problems through parenting behaviors, such as guilt-based control and unsupportive reactions [39].

Children in stressful family environments may have a heightened risk of being bullied, as discussed in relation to the correlation between bullying victimization and parent–child attachment. In this regard, the spillover effect is a critical factor in comprehending how parental marital quality influences bullying victimization through parent–child attachment. Based on these insights, it is reasonable to hypothesize that parent–child attachment mediates the relationship between parental marital quality and bullying victimization through the spillover effect.

Alternatively, the compensatory hypothesis posits that parents may attempt to mitigate a low level of marital quality by providing their children with an excessive amount of attention [34]. While this may reinforce the parent–child relationship, it can also result in emotional difficulties that affect the child’s social behavior and development, potentially increasing their susceptibility to bullying. Furthermore, in marriages that are characterized by harmony, the presence of children may be perceived as a disruption to the marital relationship, which increases the chances of parent–child conflict. Since empirical research has rarely provided evidence in favor of the compensating theory, we concentrate on the spillover effect as the most probable explanation. Given the distinct influences of mothers and fathers on child development, we decided to separate father–child and mother–child attachment in our hypotheses to explore potential differences. Accordingly, we hypothesize that

**H3a:**  *High levels of parental marital quality enhance the development of mother–child attachment.*

**H3b:**  *High levels of parental marital quality enhance the development of father–child attachment.*

**H4a:**  *Mother–child attachment mediates the relationship between parental marital quality and bullying victimization.*

**H4b:**  *Father–child attachment mediates the relationship between parental marital quality and bullying victimization.*

### 1.3. Child Gender as a Moderator

Gender plays a significant role in emotional expressions. Cultural beliefs and expectations dictate that girls are expected to exhibit cheeriness or sadness, while boys are expected to be strong and composed, displaying anger when necessary. Such feelings are mirrored in idioms like “little girls are made of sugar, spice, and everything nice” and “boys don’t cry” [40]. Boys tend to exhibit more externalizing emotions than females during middle childhood and toddler/preschool age, while at the adolescent stage, boys tend to exhibit fewer externalizing emotions [40]. Also, girls tend to form stronger emotional bonds with their parents and experience greater parental regulation compared to boys [41], which may make them more dependent on their parents during times of parental marital fluctuation and more likely to express emotions. Furthermore, girls are more likely to express emotions such as happiness, sadness, and anxiety [42]. When bullying occurs, the ability to understand and express one’s emotions facilitates quicker recovery from psychological distress [43].

In addition, parents may treat daughters and sons differently. Depending on the child’s gender, mothers and fathers changed their parenting styles [44]. Russell et al. (2003), for instance, found that whereas parents of boys reported more authoritarian parenting, parents of girls reported more authoritative parenting [45]. In terms of discipline, Mehlhausen-Hassoen (2021) found that fewer daughters than sons suffered corporal punishment, and far fewer daughters suffered from both parents. The perceived parent–child relationship was notably negatively affected by corporal punishment [46]. Furthermore, Morawska (2020) revealed that parents used different objects, socialization methods, vocalizations, and playing approaches with their children. Variations in child vocalization, emotional expressions, pain reactions, compliance, object manipulation, and hostility were linked to this unequal parenting based on child gender [47].

Gender differences also manifest in the context of school bullying, where girls are more inclined to be victims, and boys are more inclined to be the perpetrators [25,48]. Thus, it is reasonable to expect that the paths in the mediation model will be moderated by child gender. Accordingly, we hypothesize that

**H5a:**  *Child gender moderates the mediating effect of mother–child attachment in the relationship between parental marital quality and bullying victimization.*

**H5b:**  *Child gender moderates the mediating effect of father–child attachment in the relationship between parental marital quality and bullying victimization.*

### 1.4. The Current Research

**Figure 1:**
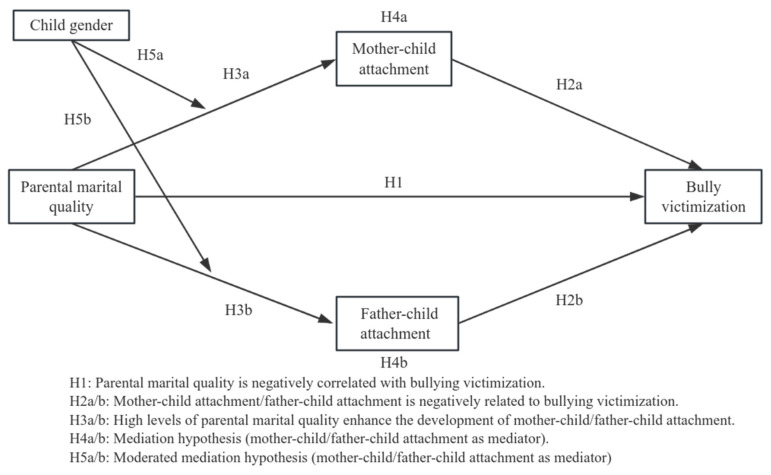
Conceptual model.

It is still not well understood the direct correlation between school bullying victimization and parental marital quality. Most research has concentrated on personal elements, paying little attention to the possible mediating and moderating processes engaged. For instance, although studies have indicated that low marital quality might cause behavioral issues in children, the mediation impact of parent–child attachment and the moderating effect of child gender have not been completely investigated. Given these gaps, the present study aims to explore the correlation between parental marital quality and school bullying victimization, with an emphasis on the mediating role of parent–child attachment and the moderating role of child gender. The theoretical framework is presented in Figure 1.

## 2. Materials and Methods

### 2.1. Participants and Procedure

This study gathered the data from three primary schools in suburban Beijing, China. The participants included students and their parents. Parents and children were each given their questionnaire as part of the survey. Mothers completed the parent questionnaire, while children filled out the child questionnaire with their parents’ permission. The child questionnaire assessed children’s experiences of bullying victimization, as well as their perceptions of father–child and mother–child attachment quality. The parent questionnaire evaluated parental marital quality. A total of 425 parent–child pairs were invited to participate. After excluding responses with missing information, 358 valid parent–child pairs remained, resulting in an 84.2% response rate. The ages of children ranged from 8 to 13 years old (M = 10.561, SD = 0.956). The characteristics of participants are given in Table 1.

### 2.2. Materials

#### 2.2.1. Bullying Victimization

Bullying victimization was measured using student questionnaires from the Child Development Project (CDP), conducted by the Developmental Studies Center (DSC) in the United States. Previous studies have used this scale with Chinese students [49,50]. The scale was completed by children and consisted of six items (e.g., “Did someone make fun of you, call you names, or insult you?”). Responses were rated on a five-point scale: never (1), 1–2 times (2), 3–5 times (3), 6–9 times (4), and 10 or more times (5). The total score for bullying victimization was calculated by summing the z-scores of the six items and then computing the average, with higher scores reflecting greater levels of bullying victimization. The confirmatory factor analysis indicated that the scale exhibited an acceptable model fit for bullying victimization (CFI = 0.976, TLI = 0.956, SRMR = 0.029, RMSEA = 0.066). The Cronbach’s α coefficient in the study was 0.79.

#### 2.2.2. Parent–Child Attachment

The Inventory of Parent and Peer Attachment (IPPA) is generally used to measure parent–child attachment [51]. This study used a shortened and modified version of the IPPA, developed by Buist et al. (2004) [52], which has been used with Chinese students [53]. The scale was comprised of 10 items, distributed across three dimensions: trust (3 items), communication (3 items), and alienation (4 items). All items were rated on a 5-point scale, ranging from “strongly disagree” (1) to “strongly agree” (5). The total parent–child attachment score was calculated by averaging the combined scores of the reversed alienation dimension, trust dimension, and communication dimension. Higher scores indicated greater attachment security. The confirmatory factor analysis indicated that the scale exhibited an acceptable model fit for mother–child attachment (CFI = 0.942, TLI = 0.919, SRMR = 0.049, RMSEA = 0.068) and father–child attachment (CFI = 0.945, TLI = 0.923, SRMR = 0.051, RMSEA = 0.073). The Cronbach’s α coefficients in the study were 0.77 for both mother–child attachment and father–child attachment.

#### 2.2.3. Parental Marital Quality

The Dyadic Adjustment Scale (DAS) is widely used for assessing marital quality [54]. The shortened version of the original DAS was used in the study (DAS-7) [55]. This scale has been widely used with Chinese samples [56,57]. The DAS-7 consisted of seven items evaluating dyadic consensus (3 items), dyadic cohesion (3 items), and dyadic satisfaction (1 item). Respondents rated the dyadic consensus and cohesion items on a 6-point scale, ranging from “always disagree or never” (0) to “always agree or more than once a day” (5). The satisfaction item was rated on a 7-point scale, ranging from “extremely unhappy” (0) to “perfect” (6). The total score was evaluated by summing the responses to all seven items, with higher scores indicating a greater degree of marital quality. The confirmatory factor analysis indicated that the scale exhibited an acceptable model fit for marital quality (CFI = 0.949, TLI = 0.911, SRMR = 0.052, RMSEA = 0.001). The Cronbach’s α coefficient in the study was 0.83.

### 2.3. Data Processing and Analysis

Descriptive statistics and Pearson correlation analyses were performed using SPSS version 26.0. Parallel mediation analyses and moderated mediation analyses were conducted using PROCESS v4.1 [58]. Model 4 in the PROCESS macro was used to test the parallel mediating effects of mother–child attachment and father–child attachment on parental marital quality and bullying victimization, controlling for children’s age [59]. Additionally, child gender was tested as a moderator of the mediation effects. The analysis employed a moderated mediation model (Model 7) to investigate whether child gender moderated the indirect relationship between parental marital quality and bullying victimization through mother–child attachment and father–child attachment, with children’s age included as a control variable. The bootstrap method with 5000 samples was applied to test the significance of effects in both the parallel and moderated mediation analyses, with statistical significance determined by 95% confidence intervals that did not include zero.

## 3. Results

### 3.1. Preliminary Analyses

The descriptive statistics and Pearson’s correlation analysis results for all variables addressed in the hypotheses are shown in Table 2. Parental marital quality was negatively correlated with bullying victimization. Parental marital quality was positively associated with both father–child attachment and mother–child attachment. Father–child attachment and mother–child attachment were negatively correlated with bullying victimization. No significant differences were found in bullying victimization between child genders. In addition, our study also found that 65.9% of students reported being bullied at school in the past year.

### 3.2. Test of the Mediating Effect

**Figure 2:**
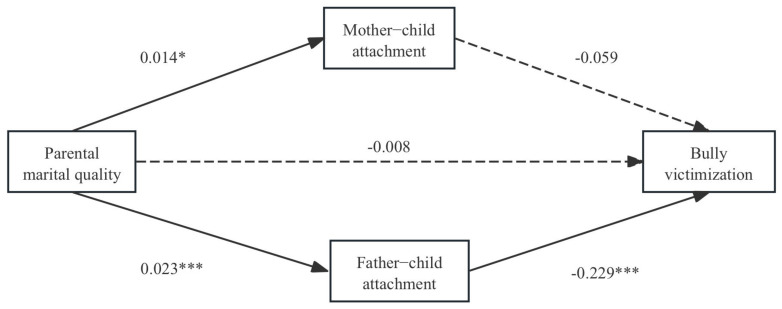
The path coefficient for the parallel mediation model. * *p* < 0.05, *** *p* < 0.001.

Model 4 of the PROCESS macro was used to test the parallel mediation effects of mother–child attachment and father–child attachment in the relationship between parental marital quality and bullying victimization. The results were presented in Figure 2, Table 3 and Table 4.

The mediation analysis, conducted using a parallel mediation model, employed four regression models to examine the mediating roles of mother–child attachment and father–child attachment in the relationship between parental marital quality and bullying victimization. In Model 1, the total effect of parental marital quality on bullying victimization was negative and significant (B = −0.014, SE = 0.006, *p* < 0.05), supporting H1 (see Table 3).

As shown in Table 3, for the mother–child attachment path, Model 2 showed that mother–child attachment was significantly and positively influenced by parental marital quality (B = 0.014, SE = 0.006, *p* < 0.05), confirming H3a. Model 4 showed that the relationship between mother–child attachment and bullying victimization was not significant (B = −0.059, SE = 0.060, *p* = 0.323), rejecting H2a. The bias-corrected percentile bootstrap method indicated that the indirect effect through mother–child attachment on bullying victimization was −0.001, with a bootstrap confidence interval of [−0.004, 0.002], constituting 5.6% of the total effect (see Table 4). This result indicates that the indirect effect through mother–child attachment was not statistically significant, as the confidence interval included zero. Therefore, the mediation effect of mother–child attachment in the relationship between parental marital quality and bullying victimization was not significant, rejecting H4a.

In contrast, for the father–child attachment path, Model 3 demonstrated that parental marital quality significantly and positively affected father–child attachment (B = 0.023, SE = 0.006, *p* < 0.001), confirming H3a. Model 4 indicated that father–child attachment significantly and negatively predicted bullying victimization (B = −0.229, SE = 0.058, *p* < 0.001), supporting H2b. The bias-corrected percentile bootstrap method revealed an indirect effect of −0.005 through father–child attachment on bullying victimization, with a bootstrap confidence interval of [−0.013, −0.000], constituting 36.8% of the total effect (see Table 4). This showed that the indirect effect through father–child attachment was significant, as the confidence interval did not include zero. Therefore, the mediation effect of father–child attachment in the relationship between parental marital quality and bullying victimization was significant, supporting H4b.

### 3.3. Moderating Effect of Child Gender

Building on the previous mediation analysis, this study aimed to investigate the moderating effect of child gender on the mediation path of parental marital quality influencing bullying victimization through mother–child attachment and father–child attachment, with child gender as the moderating variable. To examine this, a moderated mediation analysis was performed using PROCESS Model 7 with 5000 bootstrap samples. As shown in Table 5, concerning the moderating effect of child gender on the mediating role of mother–child attachment, no significant relationship was found between the interaction term of child gender and parental marital quality (B = −0.020, SE = 0.012, *p* = 0.089). This indicates that child gender did not moderate the effect of parental marital quality on mother–child attachment. Therefore, Hypothesis 5a was not supported.

**Figure 3:**
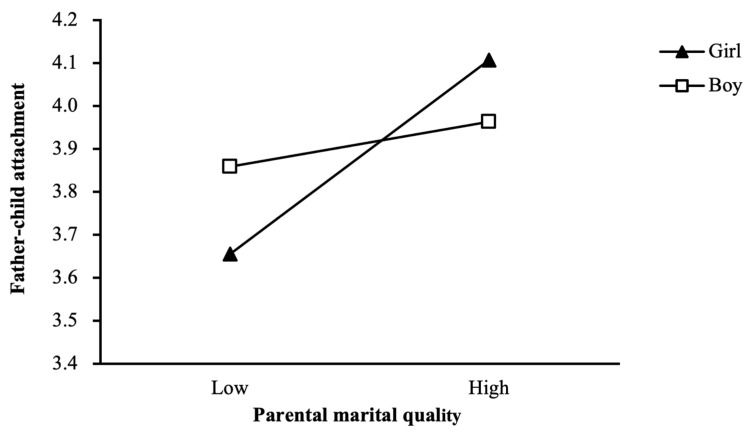
Child gender as a moderator in the relationship between parental marital quality and father–child attachment.

In contrast, regarding the moderating effect of child gender on the mediating role of father–child attachment, a significant relationship was found between the interaction term of child gender and parental marital quality (B = −0.028, SE = 0.012, *p* < 0.05). This suggested that the effect of parental marital quality on father–child attachment was moderated by child gender (see Table 5). As shown in Figure 3, the simple slope analysis showed that for girls, the simple slope was 0.036, *p* < 0.001, indicating a significant positive predictive effect of parental marital quality on father–child attachment. For boys, the simple slope was 0.008, *p* = 0.353, suggesting a non-significant positive predictive effect of parental marital quality on father–child attachment.

The significance of the conditional indirect relationship between parental marital quality and bullying victimization, mediated by father–child attachment, was examined by considering child gender as a moderator, with separate analyses for girls and boys. As shown in Table 6, the analysis revealed a significant conditional indirect effect for girls (effect = −0.008, 95% CI [−0.019, −0.001]), but no significant effect for boys (effect = −0.002, 95% CI [−0.009, 0.002]).

Moreover, the index of moderated mediation with father–child attachment as the mediator was significant (effect = 0.006), with a confidence interval that did not include zero (95% CI [0.000, 0.017]). These findings provided support for hypothesis H5b.

## 4. Discussion

This study investigated the correlation between parental marital quality and victimization from school bullying, emphasizing the mediating influence of parent–child attachment and the moderating influence of child gender. The research indicated that parental marital quality had a direct effect on victimization from bullying and that father–child attachment played a significant mediating role in this relationship. However, no mediating effect was present for mother–child attachment, which reflected the dissimilar roles that mothers and fathers play within children’s development. Additionally, child gender emerged as a significant moderator in the mediating effect of father–child attachment, with the effect being significant for girls but not for boys. This finding illustrates the value of considering gender differences when examining how parental attachment influences children’s susceptibility to bullying.

### 4.1. Hypothesis Model

In line with family systems theory, which posits that the parental marital relationship is the key subsystem that affects family dynamics and child development [17], the present study revealed a correlation between parental marital quality and children’s victimization from bullying, confirming H1. Enhancing marital quality had a protective effect, reducing the likelihood of children being bullied. This finding aligns with the dynamic nature of family systems theory, where changes in one subsystem—such as marital quality—can influence other parts of the system, including child development. These dynamics are further reflected in social learning theory, which suggests that children tend to model the conflict resolution strategies they observe in their parents [19]. When parents employ maladaptive conflict resolution strategies, children may replicate these behaviors in their peer interactions, making them more vulnerable to bullying.

Building on family system theory, we explored the role of parent–child attachment in mediating the relationship between parental marital quality and children’s bullying victimization, aiming to understand how changes in the marital subsystem can impact the parent–child subsystem and, in turn, affect children’s experiences of being bullied. While there was a strong link between parental marital quality and mother–child attachment, the mediating effect of mother–child attachment was not significant. Consequently, H2a and H4a were rejected, and H3a was accepted. The result challenges the generally accepted wisdom that women are the primary caregivers and source of emotional support for their children [60]. One possible explanation is that mothers may prioritize emotional comfort and safety. Mothers often provide comfort and emotional support, frequently using emotion-related words [61]. While emotional support is beneficial, it may not equip children with the necessary tools to handle bullying. This outcome can also be understood through the overprotective parenting theory, which asserts that overprotective parenting can impede children’s development of self-efficacy and coping skills, resulting in maladaptive psychological consequences such as anxiety and sadness, as well as increasing the likelihood that children will become victims of bullying at school [62,63,64]. A recent systematic analysis indicates that maternal overprotection may exert a more harmful effect on adolescent maladaptive functioning compared to paternal overprotection [62]. Thus, maternal overprotection may restrict the advantageous effects of the mother–child relationship to a greater extent than paternal overprotection, hindering the development of coping mechanisms and potentially heightening children’s susceptibility to bullying.

On the other hand, the father’s role in the family dynamics system was brought to light by the significant mediating effect of father–child attachment, which confirmed H2b, H3b, and H4b. Father–child attachment has a crucial influence on children’s bullying victimization. Some previous research has emphasized the critical role fathers play in fostering social competence and preventing violence [65,66]. The influence of father engagement and father–daughter relationship quality on social bullying victimizing Black females was investigated recently. The findings indicated that increased father engagement correlates with a reduced probability of social bullying victimization, but the quality of communication between fathers and daughters also affected victimization rates [67]. With fatherly care and protection acting as main predictors of bullying involvement, a recent study also confirmed the vital role of father–child attachment in determining children’s vulnerability to bullying. Particularly in children with disabilities, stronger father–child relationships served as a protective mechanism against either victimizing or bullying behavior [68]. It is now abundantly evident that fathers’ participation in the prevention of victimization is crucial.

In Chinese culture, the traditional notion of “a strict father and a benevolent mother” promotes a complementary distribution of parental roles in the family, balancing rules and feelings. Fathers frequently perform the role of the “stern father”, instructing their children on social norms and boundaries. Fathers teach their children how to resolve problems and follow social standards by establishing rules and explaining reasons, which may help prevent bullying during peer interactions. Mothers, on the other hand, take on the role of the “benevolent mother”, emphasizing emotional support and security. This traditional Chinese cultural concept, which is also consistent with earlier studies [31,32], illustrates the disparities in parental roles within the family. This cultural traditional notion also helps to explain why father–child attachment mediated marital quality and bullying victimization in our study, whereas mother–child attachment did not. The father’s “sternness” appears to be more beneficial in supporting children in confronting bullying, whereas the mother’s “benevolence”, while vital for emotional support, does not effectively help children cope with bullying.

Differences in societal expectations regarding gender roles might explain the moderating influence of child gender on the mediating effect of father–child attachment, which was particularly significant for girls but not for boys, proving H5b and disproving H5a. David and Brannon (1976) [69] identified four essential criteria of traditional masculinity: (1) “No sissy stuff,” which asserts that men should steer clear of anything that is associated with femininity; (2) “The big wheel,” which emphasizes the expectation that men will pursue success and achievement; (3) “The sturdy oak,” which suggests that men should conceal any signs of weakness; and (4) “Give ‘em hell,” which encourages men to seek adventure, even if it requires it to involve resorting to violence [69]. Boys influenced by traditional masculine ideology may rely more on their own power when confronted with violence, such as bullying. This emphasis on independence might weaken the protective role of the father–child relationship in mitigating bullying occurrences.

In contrast, girls, influenced by cultural expectations of emotional expression and nurturing, are more likely to seek emotional support and guidance from their parents when dealing with bullying. As a result, girls often exhibited stronger emotional bonds with their parents [41], which may make them more dependent on their parents during times of parental marital fluctuation and more likely to express emotions such as happiness, sadness, and anxiety [42]. Studies also suggest that father–daughter relationships play a particularly crucial role in emotional regulation, with this effect being stronger for girls than boys. Positive outcomes in emotional coping, academic performance, job accomplishments, and healthy emotional regulation are associated with father participation and positive father–daughter connections; some of these impacts continue into adulthood [70,71,72]. The capacity to express and manage emotions may empower victims of bullying and cyberbullying to utilize more extensive resources and adaptive coping mechanisms, mitigating negative emotions and enhancing positive ones to maintain better mental health and heightened life satisfaction [43]. The moderating role of child gender highlights the need to take child gender variations into account regarding parent–child attachment. Girls may rely more on their parents during family conflicts.

### 4.2. Limitations and Implications

Several limitations should be taken into account when interpreting the findings of this study. First, this study concentrated on elementary school students, a population more likely than older students to be victims of school bullying [73,74]. The risk of bullying seems to follow a “rise-then-fall” pattern that begins in elementary school [75] and typically decreases as children grow [76]. Future studies should investigate this dynamic across a wider age range since the relationship between bullying and children may change with age. Second, this study relied only on student self-reports about parent–child bonding and bullying. Self-reports are inherently subjective, and adolescents may intentionally underreport or overreport sensitive or difficult-to-recall behaviors due to social desirability concerns [77]. To provide a more comprehensive picture, future research should incorporate multi-source data, such as peer or teacher ratings. Third, in this study, mothers were the only ones who reported parental marital quality. Men and women may perceive marital quality differently [78], with men generally reporting greater levels of marital satisfaction [79]. This limitation can be further explained by the Fathering Vulnerability Hypothesis. According to this hypothesis, compared to mothers, fathers’ parenting behaviors are more susceptible to risk factors such as marital conflict [80], which might have an impact on their parent–child relationship. Future research should get a more comprehensive grasp of family dynamics by considering fathers’ perspectives. Fourth, while this study found that father–daughter attachment has a significant protective effect against becoming a victim of bullying at school, this conclusion should be interpreted cautiously due to the lack of control for other potential confounding variables (such as child temperament, family structure, or socioeconomic status) in the current analysis. Previous research has shown, for example, that disruptions in family structure, such as divorce and parental death, can change family dynamics and increase children’s susceptibility to bullying [81,82]. Incorporating these aspects into future studies may improve our understanding of the complicated dynamics. Finally, the cross-sectional design of this study may restrict causal conclusions [83]. A more profound understanding of how family dynamics affect school victimization across time might come from longitudinal studies.

Notwithstanding these limits, this study’s findings have significant theoretical and practical implications. This study presents a new perspective on how the social behavior of children is influenced by familial surroundings. More especially, it stresses the significance of parental marital quality within the family system and how parent–child attachment indirectly influences children’s sensitivity to bullying. This study enhances the application of family systems theory and social learning theory, both of which underline the connectivity of family interactions and their influence on children’s development [17,19]. Furthermore, the data add to the continuing argument between the compensation and spillover hypotheses [34]. The results provide further support for the spillover hypothesis, suggesting that marital quality can spill over into parent–child relationships, influencing the emotional security children need to cope with external challenges, such as bullying.

Building on these results, the implications for dealing with school bullying should go beyond improving parents’ influence on fulfilling children’s emotional needs. Along with offering emotional support, it is equally crucial to concentrate on enhancing children’s coping mechanisms and social adaptability skills. Encouragement of father–child connections and strengthening of father–child bonding could also help adolescents cope better, lowering their vulnerability to bullying [67]. Child gender differences should also be considered, as strengthening father–child attachment may be especially beneficial for girls.

## 5. Conclusions

This study highlighted the mediating effect of father–child attachment on the relationship between parental marital quality and bullying victimization, with child gender serving as a moderating factor. The results highlight how, especially for girls, the father–child attachment’s protective function helps to minimize the harmful consequences of parental marital conflict. These findings strengthen our knowledge of how family dynamics affect the bullying experiences of children. Practically, the study underlines the necessity of building father–child interactions and addressing children’s gender-based needs. Future studies could look at other elements influencing this link, providing a more thorough understanding of practical solutions for bullying prevention.

## Figures and Tables

**Table 1:** Sample characteristics (N = 358).

Variable	Items	Frequency (N)	Percentage (%)
Gender (Child)	Girl	172	48.0
	Boy	186	52.0
Age (Child)	8	1	0.3
	9	58	16.2
	10	94	26.3
	11	151	42.2
	12	52	14.5
	13	2	0.6
Grade (Child)	4th grade	117	32.7
	5th grade	129	36.0
	6th grade	112	31.3

**Table 2:** Descriptive statistics and correlation coefficients.

Variable	M	SD	1	2	3	4	5	6
1. Gender (Child)			-					
2. Age (Child)	10.561	0.956	0.021	-				
3. Mother–child attachment	3.973	0.709	−0.005	0.002	-			
4. Father–child attachment	3.895	0.742	0.026	0.039	0.568 **	-		
5. Parental marital quality	20.569	6.278	0.022	−0.105 *	0.118 *	0.190 **	-	
6. Bullying victimization	−0.002	0.693	0.033	−0.121 *	−0.208 **	−0.297 **	−0.116 *	-

* *p* < 0.05, ** *p* < 0.01.

**Table 3:** Regression analysis of the parallel mediation model (N = 358).

	Model 1		Model 2		Model 3		Model 4	
	Bullying Victimization		Mother–Child Attachment		Father–Child Attachment		Bullying Victimization	
	*B*	*t*	*B*	*t*	*B*	*t*	*B*	*t*
Constant	1.328	3.064 **	3.583	8.021 ***	2.933	6.358 ***	2.210	4.846 ***
Age (Child)	−0.098	−2.564 *	0.011	0.269	0.046	1.132	−0.087	−2.356 *
Parental marital quality	−0.014	−2.476 *	0.014	2.261 *	0.023	3.755 ***	−0.008	−1.451
Mother–child attachment							−0.059	−0.978
Father–child attachment							−0.229	−3.959 ***
R^2^	0.031		0.014		0.040		0.198	
F	5.748 **		2.555		7.326 ***		10.732 ***	

* *p* < 0.05, ** *p* < 0.01, *** *p* < 0.001.

**Table 4:** Bootstrap analysis of the mediation effect test (N = 358).

	Effect	Boot SE	Boot LLCI	Boot ULCI	Proportion of Effect
Total effect	−0.014				
Total indirect effect	−0.006				
Indirect effect Path 1: parental marital quality → mother–child attachment → bullying victimization	−0.001	0.001	−0.004	0.002	5.6%
Indirect effect Path 2: parental marital quality → father–child attachment → bullying victimization	−0.005	0.003	−0.013	−0.000	36.8%

**Table 5:** Moderated mediation model.

	B	SE	t	p	LLCI	ULCI
Mother–child attachment as Mediator						
Constant	3.889	0.417	9.317	0.000	3.068	4.710
Age (Child)	0.009	0.039	0.219	0.827	−0.069	0.086
Parental marital quality	0.023	0.008	2.823	0.005	0.007	0.039
Gender (Child)	−0.011	0.075	−0.152	0.880	−0.158	0.135
Parental marital quality × Child gender (H5a)	−0.020	0.012	−1.707	0.089	−0.044	0.003
Father–child attachment as mediator						
Constant	3.430	0.430	7.984	0.000	2.585	4.275
Age (Child)	0.043	0.040	1.056	0.292	−0.037	0.122
Parental marital quality	0.036	0.008	4.289	0.000	0.020	0.053
Gender (Child)	0.030	0.077	0.391	0.696	−0.121	0.181
Parental marital quality × Child gender (H5b)	−0.028	0.012	−2.251	0.025	−0.052	−0.004

**Table 6:** Conditional indirect effects.

Mediator	Conditions	Indirect Effects (Parental Marital Quality → Bullying Victimization)			
		**Effect**	**Boot SE**	**Boot LLCI**	**Boot ULCI**
Father–child attachment	Girl (child gender)	−0.008	0.005	−0.019	−0.001
	Boy (child gender)	−0.002	0.003	−0.009	0.002

## Data Availability

The original contributions presented in this study are included in the article. Further inquiries can be directed to the corresponding author.
